# Structural equation modeling of road traffic accidents based on personality dispositions 

**Published:** 2019-08

**Authors:** Ali Khanpour, Amir Abbas Rasafi, Farhad Salehian, Vahid Sadeghi

**Affiliations:** ^*a*^Imam Khomeini International University, Iran.; ^*b*^University of Tehran, Tehran, Iran.; ^*c*^Shahid Beshti University, Tehran, Iran.

## Abstract

**Background:**

According to a WHO report in the last decade, the greatest number of deaths from road traffic accidents belongs to young people in low and middle income countries and as a result of age, these incidents decrease. In response to which factor is the cause and determinant of this distinctiveness and Age-related vulnerable behavioral Difference, and Why the youth should be the most injured ones in road accidents, personality and personality differences should be considered more than any other factor; and such factors as risk taking and adventurism, high diversity and excitement, delightful pleasure, high risk readiness, and adolescent empiricism or experiences are highlighted. Among the Personality theoreticians, Hans Eysenck has given a special theoretical and explicit attention to this subject area, and has attributed personality traits to their accident-proneness. According to Eysenck, extraverts are more exposed to accident proneness and disastrous accidents because of their high need and talent for sensation seeking and being rewarded externally. The confluence of extraversion motivator with excitement and adventurous characteristics of adolescents and youths can have a high potential for driving risks and injuries in extrovert youth, especially in road modes. In the present research, 450 men and women drivers of the city of Babol in three age groups completed the Eysenck Personality Questionnaire (EPQ), Manchester Driving Behavior (MDBQ), and Attitude toward safety Approach Questionnaire.

**Methods:**

The sampling method was stratified. Structural equation model was used to assess the hypotheses and SPSS.22 and PLS2.0.3 software were used for analysis.

**Results:**

The results showed that the six factors of introversion, sensitivity to punishment, (positive) attitude toward safety, (positive) driving behavior, age and experience have inverted effect on people s accident proneness (driving accidents).

**Conclusions:**

The four factors of extraversion, neuroticism, psychoticism and sensitivity to reward also have direct impact on people accident proneness (driving accidents).

**Keywords:**

Extraversion, Introversion, Neuroticism, Psychoticism, Attitude toward safety

